# Challenges Associated With First Web Space Congenital Syndactyly Reconstruction of the Hand

**DOI:** 10.1016/j.jhsg.2026.100990

**Published:** 2026-03-26

**Authors:** Rafaela Vouza, Marina Maria Antonaraki, Panagiotis Nikolinakos

**Affiliations:** ∗ Department of Surgery, Chelsea and Westminster Hospital NHS Foundation Trust, London, UK; † School of Medicine, National and Kapodistrian University of Athens, Athens, Greece; ‡ Department of Pediatric Surgery, School of Medicine, Attikon University Hospital, National and Kapodistrian University of Athens, Athens, Greece

To the Editor:

We read with great interest the recent study by Banala et al^1^ reporting surgical approaches and outcomes in congenital first web space syndactyly. The authors have contributed to the literature by examining 69 cases over a 15-year period, addressing this rare and complex condition and providing an overview of reconstructive techniques and outcomes. In order to improve the interpretation and potential applicability of this study's results, we would like to raise several points for consideration about its specifics.

First, the retrospective nature of the study may introduce inherent limitations related to data completeness and accuracy. In addition, variation in documentation practices and thresholds for reporting complications among different surgeons may have introduced reporting bias.

Second, although the cohort of 69 cases in 56 patients represents a substantial sample for such a rare condition, the study was unable to demonstrate associations between syndactyly complexity, surgical technique, and clinical outcomes. This may reflect limited statistical power rather than the absence of clinically meaningful differences.

Furthermore, as all cases were derived from a single institution, surgical approaches may have been influenced by institutional preferences and local training practices, potentially limiting the generalizability of the findings to other centers.

In addition, a more detailed analysis distinguishing minor from major complications, their timing, and technique-specific complication profiles would enhance risk stratification and improve surgical decision-making and patient counseling, even though overall complication rates are clearly presented.

Further details regarding postoperative management would be valuable. Additional information regarding splinting protocols, hand physiotherapy, rehabilitation strategies, and duration of immobilization would be valuable, as these factors may influence functional outcomes.^2^

Moreover, functional outcomes are not assessed using validated pre- and postoperative assessment tools, limiting the objectivity of the reported surgical benefit. Finally, the study does not evaluate the influence of preoperative anatomical factors on prognosis, complication rates, or the need for revision surgery, which may be particularly relevant in this heterogeneous patient population.

We congratulate Banala et al for their contribution to the literature, expounding their methodology, and evaluating the outcomes in first web space syndactyly. Nevertheless, we believe that the findings should be interpreted cautiously in light of the limitations outlined above. We welcome the authors’ future work and eagerly await the results of further research on this interesting topic.

## Conflicts of Interest

No benefits in any form have been received or will be received related directly to this article.
